# Parkinson’s disease impairs grip force release during a sinusoidal force tracking task

**DOI:** 10.1007/s00221-026-07241-w

**Published:** 2026-02-18

**Authors:** Sara Davidson, Kenneth Learman, Anson B. Rosenfeldt, Eric Zimmerman, Jay L. Alberts

**Affiliations:** 1 Biomedical Engineering, Cleveland Clinic, Cleveland, OH USA; 2 Youngstown State University, Youngstown, OH USA; 3 Department of Biomedical Engineering, Center for Neurological Restoration, Cleveland Clinic, 9500 Euclid Avenue, ND-20, Cleveland, OH 44195 USA

**Keywords:** Force tracking, Precision grip, Force modulation, Force generation, Force release

## Abstract

The generation, modulation, and release of grip force underlie skilled manual dexterity and object interaction. Advancing age and neurological disease compromise force generation; the impact of age and pathology on the dynamic control of grip force release is unclear. The aim of this project was to determine the impact of age and Parkinson’s disease (PD) on grip force generation and release. Young adults (YA, n = 10, 18–28 years), older adults (OA, n = 10, 57–77 years), and people with PD (PwPD, n = 10, 56–75 years) completed a sinusoidal force tracking task using a precision grip with their dominant hand. Maximum grip force was not different between groups, however, OA relative to YA exhibited deficits in force generation, release and amplitude scaling. Although PwPD had global declines in force control, force release was disproportionately affected compared to force generation. The ability to scale force during its generation and release was compromised by PD. Two-point discrimination of the thumb and index fingers was impaired in PwPD and was moderately correlated with force tracking accuracy and force amplitude. While aging impacts grip force generation and release similarly during a task requiring continuous grip force modulation, PD disproportionately affects grip force release compared to generation. Force amplitude progressively decreases in older adults and PwPD. Potential sources of PD deficits include neural circuitry changes between the prefrontal cortex and striatum, impaired sensorimotor integration, and altered motor unit structure and function. Additional work is focused on identifying the neural mechanism(s) underlying impaired control and regulation of grip forces in PwPD.

## Introduction

Declines in dexterity associated with healthy aging (Hackel et al. 1992) and Parkinson’s disease (PD) (Alberts et al. 1998; Fellows et al. 1998; Gorniak et al. 2013a, b) are well documented. Impaired dexterity and micrographia are among the most bothersome PD symptoms reported (Lerner et al. 2025). Considering poor dexterity is a major contributor to transition from independence to assisted living in older adults (Ostwald et al. 1989), understanding potential changes in the control and modulation of grip forces is critical in understanding and potentially altering the impact of PD on dexterous function.

Modulation of grip forces is essential for successful and efficient completion of manual tasks. For example, handwriting requires small, frequent adjustments in grip force as letters are formed; disruptions in these force adjustments contribute to PD micrographia (Teulings & Stelmach 1991). In healthy young and older adults, grip force modulation during functional tasks is largely preserved and utilizes feedforward motor control, as sensory input and past experience inform the timing of changes in grip force as the movement is executed (Johansson & Edin 1993). In general, grip force is tightly coupled with load force demands, preventing object slippage while not exerting excessive forces (Flanagan et al. 1995; Westling and Johansson 1984). In contrast, people with PD (PwPD) rely more heavily on feedback to control grip forces, which is corrective rather than anticipatory and results in a stair stepping increase in grip forces that is uncoupled with load forces (Alberts et al. 1998; Gorniak et al. 2013a, b).

Dysfunctional control of forces among PwPD has also been observed in pursuit tracking, where participants are provided real-time visual feedback of their grip force in relation to a target—a task requiring control of hand musculature, sensorimotor integration, and executive functioning (Inzelberg et al. 2008). Compared to healthy older adults (OA), PwPD are less accurate during pursuit tracking even when controlling for tremor (Pradhan et al. 2010; Spirduso et al. 2005) and rely more heavily on visual feedback (Fellows et al. 1998), further supporting PD-related feedforward control deficits. Unlike OA, PwPD have similar tracking errors for regular sine waves and irregular, unpredictable sine waves, suggesting that PwPD exhibit impaired task motor learning and approach each sine wave as though it is unpredictable (Flowers 1978).

Despite grip force release being the terminal component of functional tasks (e.g. returning a toothbrush to the holder, hanging a towel on a hook, setting a cup on the counter), PD force modulation research has predominately focused on the generation and maintenance of forces (Stelmach et al. 1989; Stelmach and Worringham 1988; Vaillancourt et al. 2001). Limited data exist on how PD impacts grip force release (Gordon 1998; Jordan et al. 1992; Kunesch et al. 1995). We previously reported on a discrete ramp-hold-release grip force tracking task, which is among the few studies that have examined the effects of PD on grip force release and generation separately. Compared to matched healthy controls, PwPD had greater impairment when releasing grip force than when generating force, as evidenced by increased trial-by-trial variability during release (Davidson et al. 2025). Relying on different neural processes, force generation requires progressive recruitment of motor units while force release requires de-recruitment of motor units (Spraker et al. 2009). Even among healthy young adults (YA), there is increased force variability and error during force release compared to force generation (Park et al. 2016). Thus, studies evaluating force generation only or combining force generation and release are likely overlooking nuances in central nervous system changes related to PD and aging.

The primary aim of this project was to understand the effects of advancing age and PD on the ability to *continuously* modulate the generation and release of grip force during a sine wave tracking task. It was hypothesized that OA would exhibit increased impairment in grip force release compared to young healthy controls (YA) and that PwPD would be more impaired than YA and OA. The secondary aim was to determine if force release was impacted differently from force generation across groups. Based on the relative difficulty of force release compared to generation, particularly for PwPD, it was hypothesized that PwPD would show greater impairment in force release than generation. An exploratory aim was to characterize differences in sine wave tracking strategies among the three groups.

## Materials and methods

### Participants

The study was approved by the Cleveland Clinic Institutional Review Board and all participants completed the informed consent process. All participants had normal hearing and normal or corrected vision and were free of upper extremity musculoskeletal deficits that could impact precision grip. The YA and OA were free of known neurological disorders. Participants with PD were excluded if they had any other neurological disorders or if they were unable to withhold anti-parkinsonian medication for 12 h.

### Instrumentation

Grip force (Fz) data were collected with a Mini-40 force-torque transducer (ATI Industrial Automation, Garner, NC, USA) within a custom aluminum housing. Maximum voluntary contraction (MVC) data were collected at a sampling rate of 100 Hz and sine wave tracking data were collected at 30 Hz with a resolution of 0.01 N via a custom Python 3 script. Instantaneous real-time visual feedback of grip force was displayed on a computer monitor positioned at the participant’s midline. Offline data analyses were performed in MATLAB R2021a.

### Task and procedures

Participants were seated at a table with the force transducer positioned at their midline on the table. All force tracking and MVC testing was performed with precision grip of the dominant hand only. Three MVC trials were performed with one- to two-minute rest breaks between trials. The maximum force achieved was used to create a participant specific sine wave for tracking. For the force tracking task, the participant’s produced force was displayed relative to the target force on a computer monitor. The display showed ~ 5 s of the previous force output overlaid on the target force and ~ 4 s of the upcoming target trajectory. The display moved smoothly from right to left such that the current instantaneous force output was always in the same horizontal plane on the computer monitor. Participants were instructed to “follow the target line as closely as possible” and were encouraged to achieve the minimum and maximum forces.

Following one to two familiarization trials, participants completed 10 sine wave tracking trials: a 0.2 Hz sine wave for 32 s. The minimum and maximum values of the sine wave were set to 10% and 30% of the participant’s MVC, respectively. Following 10 trials, self-reports of hand fatigue based on a visual analog score from 0 to 100 were collected, with 100 indicating maximal fatigue.

Sensory testing of the dominant thumb and index fingers was performed using Semmes–Weinstein Monofilaments Test (SWMT, a test of light touch sensitivity) and two-point discrimination (TPD, a test of sensory nerve density) (Bell-Krotoski et al. 1993). The outcome for SWMT is the size of the thinnest monofilament that the participant can reliably sense on the palmar surface of the distal phalanx with their eyes closed. For TPD, the outcome is the smallest distance (in mm) between two points that a participant can reliably perceive as two distinct points on the palmar surface of the distal phalanx with their eyes closed. For both tests, smaller values indicate better sensation.

For PwPD, motor symptoms were characterized using the MDS-Unified Parkinson’s Disease Rating Scale (MDS-UPDRS-III).

### Data reduction

Linear interpolation was applied to the raw force tracking data to ensure uniform samples of 30 Hz, then a 2nd order Butterworth filter with a 12 Hz cutoff was applied to smooth the data. The first two seconds of the force tracking trial were removed to account for initial force adjustments. Data were separated into force generation and release phases based on the slope of the target force. Relative Root Mean Squared Error (RRMSE) and Percent Time Within 5% of Target Range (%TWR) were calculated for each phase.

#### Relative root mean squared error

Error during force tracking was quantified using RRMSE (formula 1), which normalizes error based on the maximum force values. F_T_(*t*) is the target force, F_0_(*t*) is the actual force produced, and *T* is the time of the trial. A lower RRMSE represents a smaller error.

1 $$ {\mathrm{RRMSE}} = \sqrt {\frac{1}{T}\mathop \sum \limits_{t = 0}^{T} \frac{{\left( {F_{0} \left( t \right) - F_{T} \left( t \right)} \right)^{2} }}{{\max \left( {F_{T} } \right)^{2} }}} $$

#### Percent time within 5% of target range

Accuracy was quantified by calculating the percentage of time spent within ± 5% of the target force. An increased %TWR represents increased accuracy. While RRMSE better characterizes large deviations from the target force, %TWR is more sensitive to smaller deviations.

#### Least square fit metrics

Using the lsqcurvefit function in MATLAB R2021a, an equation for the best fit sine wave was calculated for each trial by minimizing the sum of the squared differences from the participant's force production. This equation is the closest approximation of the participant’s actual sine wave produced and was compared to the equation for the target sine wave for that individual. This allowed calculation of percent change in force amplitude, frequency, intercept, and phase shift compared to the target output. Briefly, the force amplitude is one-half of the total height of the sine wave (i.e. (maximum force – minimum force)/2)), frequency is the number of cycles per second, intercept is the vertical shift, and phase shift is horizontal shift (with negative values indicating lagging behind the target and positive values indicating leading ahead of the target).

### Statistical analysis

Normality was assessed visually with Q-Q plots of the residuals. Variables with non-normal distribution of residuals were considered for data transformation, then rechecked for normality. Effects of group on MVC, sensation, and fatigue were assessed with ANOVA tests (or Kruskal–Wallis tests for the sensation tests, which were ordinal data). To assess the impact of age and PD on force tracking, separate group x phase ANOVA models were run for RRMSE and %TWR. In the event of an interaction, several post-hoc contrasts were run. To answer the primary research question (the impact of age and PD on force generation and release), separate post-hoc contrasts for generation and release were conducted. To explore the secondary research question (do age and PD affect release differently than generation), specific post-hoc contrasts that examined the difference between generation and release phases among groups (i.e. generation minus release) were conducted. To characterize group differences in force tracking strategies, least square fit metrics (force amplitude, frequency, phase shift, and intercept) were assessed with ANOVA tests (or Kruskal–Wallis tests for non-normal distribution). All post-hoc contrast p-values were adjusted with Holm-Bonferroni corrections for multiple comparisons. Spearman’s rho correlations were used to assess relationships between sensory outcomes and force tracking performance across groups. All statistics were performed in R (version 4.3.2).

## Results

Data from 34 YA, OA, and PwPD were collected; four individuals were removed from analysis due to equipment calibration error (n = 2), undisclosed essential tremor (n = 1), and an undisclosed pre-existing dominant hand injury (n = 1). Analysis included 30 individuals: 10 YA, 10 OA, and 10 PwPD. All PwPD were tested OFF anti-parkinsonian medication, operationally defined as 12 or more hours since the last dose of medication. Demographic information for all groups is provided in Table 1.

**Table 1:** Participant demographics

	YA (n = 10)	OA (n = 10)	PwPD (n = 10)
Age (y)	22.6 ± 2.8	67.7 ± 7.2	67.4 ± 6.6
Male sex (versus female), n	4 (40%)	4 (40%)	4 (40%)
*Race, n*			
African American	1 (10%)	1 (10%)	2 (20%)
White	9 (90%)	9 (90%)	8 (80%)
Dominant hand right (versus left), n	7 (70%)	10 (100%)	9 (90%)
Dominant side is more affected, n	–	–	6 (60%)
MDS UPDRS-III score	–	–	29 [23, 35]
Years since PD diagnosis	–	–	6.6 ± 3.4
Levodopa equivalent daily dose	–	–	511 ± 238
MVC, dominant side (N)	54.9 ± 9.2	52.1 ± 14.3	52.9 ± 19.7
*SWMT (dominant side)*			
Index	2.83 [2.83, 2.83]	2.83 [2.83, 2.83]	3.61 [2.83, 3.61]
Thumb	2.83 [2.83, 2.83]	2.83 [2.83, 3.61]	3.61 [2.83, 3.61]
*TPD (dominant side)*			
Index (mm)	3.00 [2.00, 3.00]	3.50 [2.25, 4.00]	5.00 [3.25, 5.75]
Thumb (mm)	2.50 [2.00, 3.00]	4.00 [3.25, 4.00]	4.50 [3.25, 5.00]

Summary statistics presented as mean ± standard deviation for normally distributed data, median [Q1, Q3] for skewed data, or n (%) for categorical data. YA, young adults; OA, older adults; PwPD, people with Parkinson’s disease; MVC, maximum voluntary contraction; N, Newtons; SWMT, Semmes–Weinstein monofilament test; TPD, two-point discrimination

### Age and PD increase error during force generation and release

Due to non-normal distribution, generation and release phase RRMSE were log-transformed. Repeated measures ANOVAs to compare group effects of release and generation phase RRMSE showed significant effects of group (F_2,27_ = 19.18, *p* < 0.0001) and phase (F_1,27_ = 4.44, *p* = 0.04), but no interaction. Post-hoc testing for main effect of group found significant differences between all groups (OA vs PwPD *p* = 0.01, YA vs OA *p* < 0.01, and YA vs PwPD *p* < 0.0001), with age and PD progressively worsening overall RRMSE. All groups were collapsed to assess the main effect of phase, which found that RRMSE during release was 4% better than generation (95% CI [0%, 8%]). As there was a non-significant interaction effect, no further analysis was completed.

### PD shows greater impairment in release accuracy than generation

**Fig. 1:**
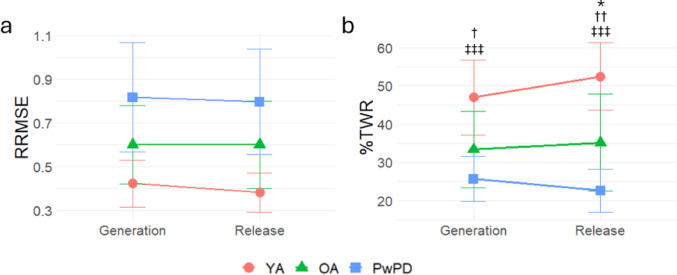
Line plots of generation and release phase **A** relative root mean squared error (RRMSE), where higher values indicate greater error, and **B** percent time within target range (%TWR), where higher values indicate greater accuracy. Note that the %TWR interaction is driven by the release phase. The PD group exhibited the greatest error and worst accuracy of the three groups. Error bars represent standard deviation. * OA versus PwPD, † YA versus OA, ‡ YA versus PwPD; * *p* <.05, ** *p* < .01, *** *p* < .001

Repeated measures ANOVA for %TWR showed significant group (F_2,27_ = 21.65, *p* < 0.0001) and interaction effects (F_2,27_ = 6.41, *p* < 0.01). For generation %TWR, there were significant differences between YA and OA (*p* < 0.02) and between YA and PwPD (*p* < 0.001), but not between OA and PwPD (*p* = 0.21). There were significant differences between all groups for release %TWR (OA vs PwPD *p* < 0.03, YA vs OA *p* < 0.01, YA vs PwPD *p* =  < 0.0001), with age and PD progressively worsening accuracy. Post-hoc comparisons of group %TWR generation minus release were run. The results suggest this group x phase interaction is driven by reduced accuracy in the release phase in PwPD compared to YA (*p* < 0.01); the other comparisons were not significant (OA vs. PwPD *p* = 0.11, YA vs. OA *p* = 0.13). See Table 2 for a summary of mean RRMSE and %TWR values for each group and phase and Fig. 1 for line plots.

**Table 2:** Means and standard deviations for RRMSE, %TWR, and least square fit metrics, and results of ANOVAs (or Kruskal–Wallis for intercept)

Variable	YA	OA	PwPD	Group *P* Value	Phase *P* Value	Interaction *P* Value
*RRMSE*						
Generation phase	0.42 (0.11)	0.60 (0.18)	0.82 (0.25)	** < 0.0001**	**0.04**	0.20
Release phase	0.38 (0.09)	0.60 (0.20)	0.80 (0.24)			
*%TWR*						
Generation phase	46.98 (9.88)	33.38 (9.94)	25.67 (5.85)	** < 0.0001**	0.16	< 0.01
Release Phase	52.44 (8.83)	35.16 (12.72)	22.63 (5.57)			
*Least square fit (% change)*						
Force amplitude	− 16.57 (4.09)	− 26.55 (9.36)	− 39.25 (11.54)	** < 0.0001**	**–**	**–**
Frequency	0.17 (0.12)	0.20 (0.43)	− 0.37 (0.24)	** < 0.001**	**–**	**–**
Phase Shift	− 0.50 (0.96)	− 1.41 (2.54)	0.94 (2.45)	0.06	**–**	**–**
Intercept	− 0.61 (3.05)	− 2.07 (8.03)	− 5.11 (10.19)	0.80	**–**	**–**

Bold faced text indicates statisticalsignificance (*p* < 0.05).

Least square fit values represent percent change from the target sine wave parameters

### Decreased force amplitude with age and PD

**Fig. 2:**
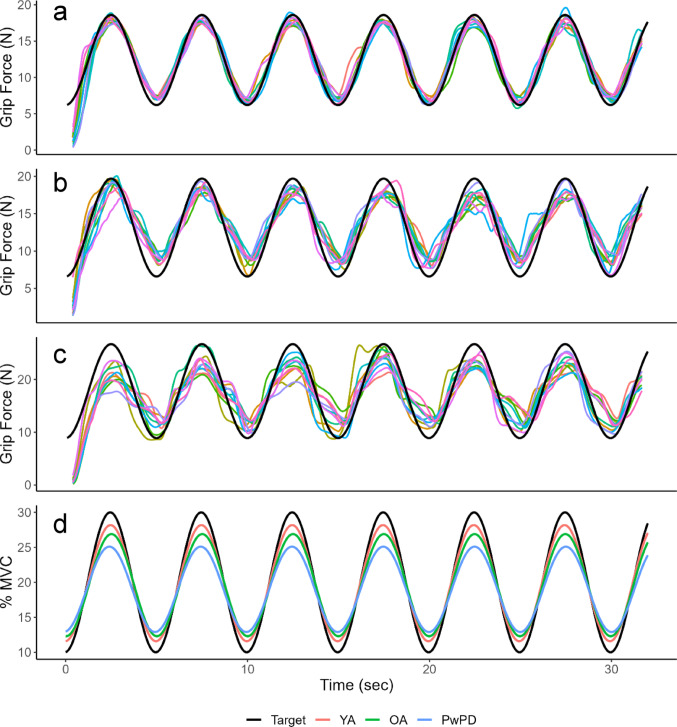
Representative trials from **A** a YA, **B** an OA, and **C** a PwPD. The black line indicates the target force, the upper and lower bounds of which were fixed at 10–30% of the participant’s maximum voluntary contraction (MVC). The colored lines represent the participant’s actual force (20-sample rolling avg) for all 10 trials. Progressive decline in amplitude and performance is seen with healthy aging and PD. **D** Representative sine wave traces based on mean least square fit values for each group, with the black line indicating normalized target force. Note the clear group differences in amplitude

Analysis of least square fit metrics revealed significant group effects for force amplitude (F_2,27_ = 16.31, *p* < 0.0001) and frequency (F_2,27_ = 11.76, *p* < 0.001) but not phase shift or intercept. Post-hoc analyses found significant differences among all groups for force amplitude (OA vs PwPD *p* < 0.01; YA vs OA *p* = 0.02; YA vs PwPD *p* < 0.0001), with age and PD progressively worsening force amplitude. Group differences for frequency were not significant between YA and OA, but PwPD had significantly slower frequencies than OA and YA (both *p* < 0.001). See Table 2 for a summary of least square fit outcomes. Based on the mean least square fit values for each group, representative sine waves were constructed for each group and compared to the target sine wave profile (see Fig. 2D).

### No group differences for maximum grip force or fatigue

There were no significant differences between groups for MVC (Table 1). There were no significant group differences in fatigue following the tracking tasks with mean (SD) values of 36.2 (19.0) for YA, 21.2 (17.3) for OA, and 30.5 (23.7) for PwPD.

### Sensation different between groups and correlated with tracking performance

Kruskal–Wallis tests showed significant main effects of group for SWMT of the index (χ^2^(2) = 8.24, *p* = 0.02), TPD of the index (χ^2^(2) = 8.55, *p* = 0.01), and TPD of the thumb (χ^2^(2) = 9.14, *p* = 0.01), but not of SWMT of the thumb. Post-hoc testing of SWMT of the index confirmed significant group differences between OA and PwPD (*p* = 0.04) and between YA and PwPD (*p* = 0.03), with PwPD exhibiting worse sensation than the control groups. Young adults had a significantly better TPD than PwPD for both the index and thumb (both *p* < 0.05). See Table 1 for a summary of sensation outcomes by group.

Spearman’s rho revealed weak to moderate relationships between index SWMT and phase shift (*r*_*s*_(28) = − 0.47, *p* = 0.02); between index TPD and generation %TWR (*r*_*s*_(28) = -0.47, *p* = 0.02), release %TWR (*r*_*s*_(28) = − 0.44, *p* = 0.02), force amplitude (*r*_*s*_(28) = -0.42, *p* = 0.03), and frequency (*r*_*s*_(28) = − 0.35, *p* = 0.007); and between thumb TPD and generation %TWR (*r*_*s*_(28) = − 0.47, *p* = 0.03), release %TWR (*r*_*s*_(28) = − 0.48, *p* = 0.008), and force amplitude (*r*_*s*_(28) = − 0.47, *p* = 0.02).

## Discussion

This study examined the effects of age and PD on grip force modulation during a sine wave pursuit tracking task. While healthy aging appears to similarly impact the modulation of grip force generation and release, PD differentially impairs force release more than generation, supporting the hypothesis that PD impairs grip force release beyond what is associated with healthy aging. Specifically, PwPD had greater impairments in the accuracy of force release than generation (i.e. generation %TWR minus release %TWR was significantly different in PwPD compared to YA) even though overall error (i.e. RRMSE) was similar between phases. While RRMSE is sensitive to large deviations from the target force, %TWR quantifies consistency of accurate performance and not the magnitude of deviations. An interaction effect for %TWR but not RRMSE suggests that the PwPD had more *sustained* error during force release than during generation, even if overall error was similar. This suggests that PwPD had more difficulty correcting deviations during force release; thus, PD may disproportionally impair accurate control of force release compared to generation. Additionally, PwPD tracked the sine wave at a significantly slower frequency than the control groups (although overall change in frequency was extremely small for all groups), and force amplitude progressively decreased with age and PD.

We previously reported on this same cohort’s performance during a discrete ramp-hold-release force tracking task (Davidson et al. 2025, 2024). When comparing OA and PwPD, the ramp task revealed greater PD-related impairment in trial-by-trial variability during the release phase than generation (Davidson et al. 2025). The possible PD-related differential degradation of force release compared to generation is further supported by the reduced ability of PwPD to maintain accuracy during the release phases of the current sine wave task. An unexpected finding in both studies was that the YA were slightly more accurate during force release than generation regardless of task. As our primary research question was not to compare within-group performance on generation and release, formal statistical testing was not completed in YA to determine the statistical power of this observation. Regardless, this observation conflicts with existing literature that reports force release error and variability are greater than generation (Naik et al. 2011; Ohtaka and Fujiwara 2016; Park et al. 2016; Patel et al. 2019), with few exceptions (Ebisu et al. 2022; Spraker et al. 2009). The precise reason underlying better force release than generation in YA is unclear; however, differences in task demands associated with a precision grip compared to power grip, ankle dorsiflexion, or other configurations may be contributing to the differences. Further, other studies comparing force generation and release have used a variety of task paradigms, with some examining rapid force release and others incorporating varying rates of controlled release. The result that sensation capability was correlated with accuracy during the sine wave generation and release phases suggests that sensory input may play a key role in the current findings.

In the current study, force tracking accuracy and force amplitude were moderately correlated with TPD of both digits. Two-point discrimination tests the density of Merkel cells (Dellon 1984), which are responsive to light touch, including the velocity of touch (Abraira and Ginty 2013). As the velocity is continually changing throughout a sine wave, input of the velocity of touch is critical for feedback control, but impairments would be most detrimental during the peaks and troughs of the sine waves, where velocity is changing the most rapidly. All groups displayed diminished force amplitude compared to the target, suggesting difficulty with achieving these rapid changes in velocity. The YA group performed the best on the sensation tests and was closest to achieving the target amplitude, followed by the OA, then the PwPD. That the OA group demonstrated a similar, although blunted, increase in accuracy during sine wave release compared to generation suggests that OA utilize a feedback control process similar to YA during this task, although it is compromised by the aging process. In contrast, the PD group had worse accuracy during release than generation, pointing to an inability to appropriately correct deviations from the target and suggesting impaired feedback control. Sensation deteriorates with age (Shaffer and Harrison 2007) and further with PD (Conte et al. 2013). While sensory deficits related to healthy aging include decreased density and size of myelinated sensory receptors, including Merkel cells (Shaffer and Harrison 2007), PD-related deficits may be driven by noisy sensory input due to dopaminergic denervation of the basal ganglia (Conte et al. 2013). Thus, group differences may be partly driven by the quality of sensory input available to inform feedback processes. However, the role of other age- and PD-related changes, such as neuromuscular changes, should also be considered.

For example, OA have a less efficient motor unit de-recruitment pattern compared to YA, possibly due to co-contractions when releasing force (Kamen and De Luca 1989). Motor unit remodeling, such as increased size and decreased number of motor units, is also seen with healthy aging and is exacerbated with PD (Kelly et al. 2018). It is possible that PD impairs the efficiency of motor unit de-recruitment more than recruitment, resulting in greater deficits in controlled force release or reduced ability to integrate feedback control in a timely manner. Future studies could consider the role of motor unit structure and function during dexterous tasks among PwPD. Additionally, stress is known to influence the control and coordination of grip force modulation (Wagner et al. 2015; Sahar et al. 2023) and may be a confounding factor. A limitation of the current study was the lack of a formal or informal measure of stress, which may differ across the three groups. Studies are planned to systematically manipulate and measure grip force control in these groups to understand the interplay between grip force modulation, dual-tasking and stress.

Force tracking requires continual attention to monitor the target and force produced. In contrast to healthy OA, PwPD appear to use nearly all of their available attentional resources during a simple force tracking paradigm (Hocherman et al. 2004). For PwPD, the ability to keep pace with the target is the most affected with increased attentional demands. Thus, the high level of attention required to track the sine wave in the current study may have contributed to the reduced frequency seen in PwPD. Notably, the reduction in frequency was a fraction of a percentage in the PD group and although statistically significant, it is unclear if the reduction is functionally meaningful. The prefrontal cortex (PFC) is involved with attention (Hocherman et al. 2004), primarily outputs to the striatum (Haber 2016), and is directly affected by PD-related dopaminergic changes that disrupt control of the striatocortical loop (Hocherman et al. 2004). Interestingly, the PFC has increased activity with eccentric versus concentric contractions (Kwon and Park 2011) and with force release versus generation (Spraker et al. 2009), which could be reflective of increased cognitive demands of eccentric/releasing forces (Kwon and Park 2011). Changes in the neural circuitry between the PFC and striatum could explain the increased difficulty PwPD had maintaining accuracy during force release compared to generation.

The ability to determine the neural mechanisms behind age- and PD-related impairments is limited by the lack of a mechanistic aim in the current study. Although this study was also limited by a small sample size with large within-group variability, the significant group differences observed here are promising and justify a larger study with mechanistic elements.

This study found that while healthy aging impaired force generation and release similarly, PwPD had greater impairment in accuracy during force release than generation. Force amplitude during tracking was diminished with age and further compromised with PD. Possible explanations for the impaired sinusoidal task performance in PwPD include neural circuitry changes between the prefrontal cortex and striatum, impaired sensorimotor integration and feedback control, and altered motor unit structure and function. Future studies should assess relationships between cognition, sensation, and force tracking in PwPD. To better understand and potentially treat manual dysfunctions in OA and PwPD, the motor control of force generation and release should be appropriately parsed from one another.

## Data Availability

The corresponding author will provide datasets from the current study upon reasonable request.
